# Characteristics of Biologically Active Compounds in Cornelian Cherry Meads

**DOI:** 10.3390/molecules23082024

**Published:** 2018-08-14

**Authors:** Kinga Adamenko, Joanna Kawa-Rygielska, Alicja Z. Kucharska, Narcyz Piórecki

**Affiliations:** 1 Department of Fermentation and Cereals Technology, Faculty of Food Science, Wroclaw University of Environmental and Life Sciences, Chełmońskiego 37, 51-630 Wrocław, Poland; joanna.kawa-rygielska@upwr.edu.pl; 2 Department of Fruit, Vegetable and Plant Nutraceutical Technology, Faculty of Food Science, Wroclaw University of Environmental and Life Sciences, Chełmońskiego 37, 51-630 Wroław, Poland; alicja.kucharska@upwr.edu.pl; 3 Arboretum and Institute of Physiography in Bolestraszyce, 37-700 Przemyśl, Poland; narcyz360@gmail.com; 4 Faculty of Physical Educaiton, University of Rzeszów, Towarnickiego 3, 35-959 Rzeszów, Poland

**Keywords:** mead, *Cornus mas* L., fermentation, iridoids, polyphenols, antioxidative activity

## Abstract

In this study, we investigated the effect of Cornelian cherry cultivars differing in fruit color (“Yantaryi”—yellow fruits, “Koralovyi”—coral fruits, “Podolski”—red fruits) on physicochemical characteristics, antioxidative properties, and contents of iridoids and polyphenols in meads with the addition of juices made of their fruits. “*Trójniak*” type meads (1:2 honey to water volume) were manufactured from multifloral honey, to which Cornelian cherry fruit juice was added before fermentation. Concentrations of individual iridoids and polyphenols were determined using HPLC analysis with a thermostat refractometric detector, model RID-10A. The total polyphenol content was determined based on testing with Folin–Ciocalteu (F-C) reagent, whereas the antioxidative properties were determined using DPPH^•^ (2,2-Diphenyl-2-picryl-hydrazyl), ABTS^•⁺^ (2,2′-Azino-bis(3-ethylbenzo-thiazoline-6-sulfonic acid), and FRAP (ferric reducing antioxidant power) assays. Cultivar of Cornelian cherry fruits influenced both the antioxidative properties and the concentrations of polyphenols and iridoids. The highest concentration of total polyphenols (F-C), accounting for 898.7 mg gallic acid (GAE)/L, was determined in the mead with juice made of red fruits; this mead was also characterized by the strongest antioxidative capabilities measured with ABTS^•⁺^ and FRAP assays. Among the iridoids determined in the Cornelian cherry meads, loganic acid was found to prevail and its highest concentration, reaching 77.8 mg loganic acid (LA)/L mead, was determined in the mead with the coral-fruit juice. Study results indicate that Cornelian cherry meads have a high content of biologically active iridoids and phenolic acids which display valuable antioxidative properties.

## 1. Introduction

Mead is a fermented beverage produced via fermentation of honey, with alcohol content ranging from 8 to 18% *v*/*v* [1]. Honeybee honey, which is the basic raw material in mead production technology, is a valuable natural food product containing sugars, vitamins, minerals, proteins, and polyphenols, and offering a high nutritive value [2,3,4]. An increased contribution of natural antioxidants (polyphenols, vitamins) in a diet has been proved to reduce the incidence of neoplasms and cardiovascular diseases [5,6,7]; therefore, regular consumption of small amounts of mead may be a good supplement to other plant products, such as fruits and vegetables.

The content of biologically active compounds in meads and, hence, their antioxidative capabilities depend on many factors like, e.g., type of honey, heat processing of wort, and parameters of fermentation process, but also the type of additives used, i.e., herbs, spices, or fruits [8,9,10]. The latter contain multiple compounds which elicit antioxidative effects [11,12,13]. Among such fruits, special attention is owed to the fruits of the Cornelian cherry, which apart from polyphenols contain compounds from the group of iridoids that have so far been detected in few fruits only [14,15,16,17,18,19].

Furthermore, these fruits may be used in various branches of the food industry, including production of alcoholic beverages like, e.g., Cornelian cherry liquors [14,20,21]. We found no works in the available literature that would address the characteristics and properties of Cornelian cherry meads. Therefore, we have decided to investigate this product considering the valuable properties of both honey and Cornelian cherry fruits. The objective of this study was to determine the effect of Cornelian cherry cultivar used to produce fruit juice that was added to honey wort on the content of iridoid and phenolic compounds and on the antioxidative properties of the produced meads.

## 2. Results and Discussion

### 2.1. Dynamics of the Fermentation Process

Dynamics of the fermentation process is a key parameter enabling the control of its course and determination of its duration. Fermentation of the analyzed meads spanned for 14 days (Table 1). Introduction of Cornelian cherry juices in the mead-making process may have a significant effect on the fermentation dynamics. A positive effect on fermentation dynamics was observed in the case of samples with addition of Cornelian cherry juices, whose addition caused CO_2_ emission to be 10% higher than in control samples after 5 days of the fermentation process. The yeast strain did not have a big influence on the dynamics of fermentation of these meads. Roldán et al., 2011, in their research showed bee pollen to be an additive having a strong impact on the improvement of mead fermentation dynamics [22].

### 2.2. Sugars Profile; Ethanol, Glycerol, and Acetic Acid Content; and pH Value 

The concentrations of fructose and glucose in control samples were 190 and 130 g/L. In worts with the addition of Cornelian cherry juices, the amounts of fructose and glucose were 30 and 20 g/L higher. This is due to the fact that in honey and in Cornelian cherry fruit, which are basic raw materials for our mead production, the main sugars are glucose and fructose [14,23]. The fermentation process had a significant effect on the decreased concentrations of these sugars in all samples. Glucose was consumed by the yeast during fermentation of the samples with Cornelian cherry juices (MY—mead with the addition of yellow Cornelian cherry juice, MC—mead with the addition of coral Cornelian cherry juice, MR—mead with the addition of red Cornelian cherry juice) in the average amount of 83%. In the case of fructose, the extent of its reduction was affected by the yeast strain used for fermentation. In all variants, the *S. bayanus* Safspirit Fruit (SF) yeast consumed 20% more of this sugar compared with the *S. cerevisiae* Safspirit Malt (SM) strain. The aging process also influenced the reduction of the discussed sugars, but to a lesser extent. Evaluation of the carbohydrate profile during fermentation processes allows for controlling their course and determining the effect of technological parameters and substrates used on the extent of extract consumption [9,24]. Apart from that, sugar and alcohol contents affect the perception of mead sweetness, which has a significant impact on the sensory properties of these alcoholic drinks [25].

Our study demonstrated ethanol production to be affected by both the type of Cornelian cherry juice and the yeast strain used in mead manufacture. In the presented mead-making technology, alcohol production by the SF yeast was 20 g/L higher compared with the SM yeast, and more ethyl alcohol was produced in the sample with juice from red-fruit Cornelian cherry (MR). Earlier investigations by other authors also have confirmed ethanol concentration in the produced meads to be influenced by the yeast strain and additives used in mead making [1,9,22]. However, meads manufactured by other authors with the addition of supplements in the form of vitamins and mineral salts or with the addition of bee pollen contained less ethanol, i.e., at least 2% *v*/*v* less than the Cornelian cherry meads manufactured in our experiment [22,26]. 

Apart from ethanol, which is the major product of alcoholic fermentation, analyses were conducted for concentrations of by-products, like glycerol or acetic acid. After the completed fermentation process, the mean glycerol concentration in the manufactured meads reached 8.9 g/L. After the aging process, it increased by 0.5 g/L on average in all meads. High glycerol production by yeast during fermentation may be caused by ethanolic and osmotic stress. The quantity of produced glycerol may also be affected by the presence of organic acids (acetic, formic, succinic). Glycerol is of great significance to the quality of meads as it affects their sensory traits, like e.g., fullness of flavor, perception of sweetness, or smoothness [25,27]. Gomes et al. (2015) achieved a slightly lower glycerol concentration in their study, which was probably due to a substantially shorter fermentation period of honey worts [25]. Organic acids play a significant role in mead making because they influence the rate of the fermentation process as well as the microbiological stability and purity and organoleptic properties of meads [28]. No acetic acid was found in meads containing juices from Cornelian cherry fruits after completed fermentation with the use of SM yeast (MY-SM, MC-SM, MR-SM). A lack of acetic acid was reported in the variant with red-fruit juice addition also after the aging process. In the other variants, concentration of this acid increased insignificantly after the stage of aging. The enhanced synthesis of this compound is a negative phenomenon and may be induced by yeast response to the osmotic stress [25]. In their studies on the fermentation of honey worts, other authors demonstrated either higher [9] or similar concentrations of acetic acid in meads [25,29]. The acidity of meads was determined based on their pH value. Worts with 10% addition of Cornelian cherry juice had lower pH values compared with the control wort (W0), which could be due to the naturally low pH value of Cornelian cherry fruits [30]. In the subsequent technological stages of the mead-making process, pH values of meads decreased successively. Acidity is of great importance to the fermented beverages as it affects their taste and microbiological stability [8]. A significant drop in pH value may cause a decrease in the fermentation efficiency of a yeast strain [26]. Other investigations addressing the physicochemistry of meads demonstrated their pH values to be at a similar [8] or a negligibly higher level [22,26].

### 2.3. Concentration of Total Polyphenols and Antioxidative Activity

Study results demonstrate that the meads with the addition of juice from Cornelian cherry fruit had a significantly higher concentration of total polyphenols and significantly stronger antioxidative properties compared with the control meads (WM) without juice addition (Table 2). The highest total polyphenol concentration was determined in the mead with the addition of red juice (MR) fermented with SM yeast: it reached 898.7 mg GAE/L and was 30-fold higher than in the mead without juice addition (MW). A slightly lower concentration of total polyphenols was determined in the meads with coral-fruit juice, and then in the meads with yellow-fruit juice, i.e., 613.6 and 399.7 mg GAE/L, respectively.

The strongest antioxidative properties measured with the DPPH^•^ assay were assayed in the meads with the addition of juices from red and coral fruits (MR, MC), whereas a slightly lower activity measured with this method was found in the meads containing juice from yellow fruits (MY). This dependency was confirmed in the analysis of the antioxidative activity of meads conducted with the ABTS^•+^ assay. In turn, the FRAP assay showed no significant differences in antioxidative activity among the meads with the addition of juices from red fruits and also coral fruit fermented with yeast SM. The highest antioxidative activity was determined in the sample with the addition of juice from red fruits (MR) and fermented with SM yeast; it accounted for 6.2 mmol TE/mL (DPPH^•^ assay), for 7.1 mmol TE/L when analyzed with the ABTS^•⁺^ assay, and for 8.2 mmol TE/L when measured with the FRAP method and was 39-fold, 21-fold, and 8-fold higher, respectively, than in the mead without fruit juice addition (MW). A slightly lower value of the antioxidative activity was obtained in the meads with juices from coral and yellow fruits; however, it was still significantly higher than in the control variant when measured using all discussed methods. The antioxidative activity and chemical composition of meads is determined by the chemical composition of the raw material and the technology of its processing, by additives used in mead making including fruits or herbs [8,31], as well as by pigments present in both honey and additives. The beverages are rich in polyphenols that are very well bioaccessible [10]. The literature provides data on the total concentration of polyphenols and antioxidative properties measured with the DPPH^•^ and ABTS^•+^ assays in “trójniak” type meads [10]. The highest concentration of total polyphenols was determined by these authors in the mead with juice from rowanberry; however, its value was 45-fold lower compared with that measured in the mead with juice from red fruits of Cornelian cherry in our study (MR). In other studies on the antioxidant properties of mead, much lower antioxidant activity was obtained both in meads obtained from different kinds of honey—an average amount of 3.0 mmol TE/L (ABTS^•+^ assay) [8]—and in meads that differ in their production technology, where the highest antioxidant activity was 2.6 mmol TE/L (ABTS assay) [9].

### 2.4. Quantitative Identification of Iridoids, Phenols, and Hydroxymethylfurfural 

In worts and meads with the addition of juices from Cornelian cherry fruits, we identified compounds from the group of monoterpenes (iridoids), phenolic acids (hydroxybenzoic acids and hydroxycinnamic acids), and flavonoids (anthocyanins, flavonols) (Table 3 and Table 4, and Figure S1). After aging, the meads had the highest concentrations of iridoids (51.4–83.8 mg/L), followed by phenolic acids (4.6–6.0 mg/L) and flavonols (0.4–1.4 mg/L), and the lowest concentration of anthocyanins (0.0–0.2 mg/L). The latter were identified in trace amounts only in aged meads with the addition of juice from red fruits (fermented with SF and SM) and juice from coral fruits (fermented only with SM). Among the iridoids (Table 3), quantitatively determined were loganic acid (LA) and cornuside (Co), as well as the sum of loganine (Lo) and sweroside (S). Loganic acid was a predominating iridoid; its concentration constituted from 90 to 95% of total iridoids. The highest concentrations of La, Co, and S were determined in the mead with the addition of juice from the coral-fruit cultivar of Cornelian cherry (MC), which was fermented with SM yeast. The highest concentrations of S and Lo were obtained in the sample with the addition of juice from red fruits of Cornelian cherry (MR), fermented with SF yeast. Iridoids are a group of compounds whose presence has been analyzed in few fruit species only, including Cornelian cherry [14,15]; hence, they have not been identified in fruit meads investigated so far. Iridoids exert an immediate effect on the biological properties [32] and taste [14,33] of food products. Therefore, their presence in foods may be very beneficial and positively evaluated by consumers. Among the analyzed phenolic acids, prevailing turned out to be gallic acid (GA) and chlorogenic acid (CQA), while concentrations of the other phenolic acids were significantly lower (Table 3). Our results demonstrate an increase in gallic acid concentration in the subsequent stages of the manufacture of all meads with the addition of Cornelian cherry fruit juices. A similar dependency was observed for ellagic acid (EA) in the meads with juice from coral and red fruits. In the wort and mead without fruit juice addition, the only quantified compounds were *p*-coumaric acid (p-CuA) and chlorogenic acid (CQA), and the latter was detected only in the wort before beginning the fermentation process. A different group of compounds which affects both the biological properties and quality of plant-based foods is represented by phenolic compounds. Q-3-Glucuronoide (Q-3-glcr) was the only one among the quantified flavonols which was detected in all meads with the addition of Cornelian cherry fruit juices (Table 4). In turn, anthocyanins were detected only in the meads with the addition of juices from coral- and red-fruit cultivars of Cornelian cherry. The predominating anthocyanins were derivatives of pelargonidin (Pg-3-gal, Pg-3-rob); however, the highest concentration was determined in the meads with the addition of juice made of red fruits. Anthocyanins are a group of compounds characterized by a relatively low stability because they are sensitive to light and temperature and to changes in pH value, and this may be the reason for their decreased concentration in the finished product [34,35,36]. The other anthocyanins were detected only in the meads with the addition of juice from red fruits of Cornelian cherry. The phenolics profile in meads is affected by the type of mead used for fermentation and additives used in the mead-making technology including, in particular, fruit juices [8,37]. The conducted study proves that fruits of Cornelian cherry have a strong impact on the increase in concentrations of biologically active compounds of meads which exhibit valuable antioxidative properties, including iridoids that have anti-inflammatory activity and antibacterial, anti-fungal, and anti-spasmodic properties [32]. The fermentation process of meads results in a relatively rapid decrease in the pH value below pH = 3, which offers favorable conditions for the formation of hydroxymethylfurfural (HMF), the precursor of which is fructose—one of the major sugars of honey wort [38]. HMF production in meads is affected by the type and quality of honey, heat treatment of wort, and conditions of the fermentation process [39]. Results obtained in our experiment demonstrate that HMF detected in wort was derived from both honey and juice from Cornelian cherry fruits. In all experimental variants, this compound was completely degraded after the fermentation process, but reappeared after the aging process, though in quantities lower than in the initial worts. Differences in HMF concentration in various fermented beverages indicate conditions of the fermentation process to be of key significance to the production of alcoholic drinks with a low concentration of this contaminant and, hence, to the manufacture of safe and high-quality products [40].

## 3. Materials and Methods

### 3.1. Materials

#### 3.1.1. Reagent and Standard

1,1-Diphenyl-2-picrylhydrazyl radical (DPPH^•^), 6-hydroxy-2,5,7,8-tetramethylchroman-2-carboxylic acid (Trolox), 2,4,6-tri(2-pyridyl)-s-triazine (TPTZ), dimethyl sulfoxide (DMSO), FeCl_3_, acetonitrile, formic acid, sulfuric acid, sodium hydroxide, and hydroxymethylfurfural (HMF) were acquired from Sigma-Aldrich (Taufkirchen, Germany). Acetic acid was obtained from Chempur (Piekary Śląskie, Poland). Acetonitrile for HPLC was purchased from POCh (Gliwice, Poland). Loganic acid (LA), loganin (Lo), Sweroside (S), gallic acid (GA), ellagic acid (EA), 5-*O*-caffeoylquinic acid (5-CQA, chlorogenic acid), *p*-coumaric acid (*p*-CuA), quercetin 3-*O*-glucoside (Q glc), kaempferol 3-*O*-glucoside (Kf glc), cyanidin 3-*O*-glucoside (Cy glc) were purchased from Extrasynthese (Lyon Nord, France). All reagents were of analytical grade.

#### 3.1.2. Biological Material

The biological material were *Saccharomyces bayanus* Safspirit fruit (SF) and *Saccharomyces cerevisiae* Safspirit malt (SM) yeast from the Fermentis company (Lesaffre, Marcq-en-Barœul, France). Before being inoculated, dried yeast was rehydrated in distilled water at a temperature of 25 °C for 30 min.

#### 3.1.3. Raw Material

The studies used the same plant material as that described in detail in the publications by Kawa-Rygielska et al. [21].

### 3.2. Preparation of Samples

Rapeseed honey was stirred with water in the ratio of 1:2 (honey/water). The resultant wort was boiled at a temperature of 100 °C for 1.5 h, with regular stirring and skimming off the scum. Afterwards, the mixture was cooled and for all mead variants, the initial extract content in the wort was set at 34° Bx. Wort was divided into four variants (Table 5): control wort (W0) and three worts with Cornelian cherry juice in the amount of 10% of fermentation wort volume—wort with yellow juice (WY), wort with coral juice (WC), and wort with red juice (WR). Next, yeast was rehydrated in distilled water and inoculated in the amount of 0.5 g d.m./L of wort. Afterwards, calcium carbonate (0.4 g/L) and potassium phosphate dibasic (0.4 g/L) were added to the fermentation medium. The process of alcoholic fermentation was conducted at 22 °C for 14 days. The post-fermentation samples were subjected to the aging process at a temperature of 8 °C for 3 months.

### 3.3. Analytical Methods

#### 3.3.1. Fermentation Process Dynamics and Physicochemical Parameters

Dynamics of the fermentation process were controlled based on CO_2_ emission in time. To this end, the fermentation samples were weighed on a WTB 2000 scale by RADWAG company (Radom, Poland) every 24 h throughout the alcoholic fermentation. Extract content was determined with a Densito 30 PX densitometer by Mettler-Toledo company (Greifensee, Switzerland). The samples were earlier centrifuged using an MPW-351R laboratory centrifuge (2675 centrifugal force (RCF), 6000× *g*, 10 min) by MPW MED. INSTRUMENTS company (Warszawa, Poland). Extract content was measured in the resultant supernatant at a temperature of 20 °C. The pH value was measured with an MP 220 pH-meter by Mettler Toledo company (Greifensee, Switzerland).

#### 3.3.2. Sugars, Ethyl Alcohol, Acetic Acid, and Glycerol Content

The concentrations of sugars (fructose, glucose), alcohol, organic acid, and glycerol were determined by means of HPLC [41]. Centrifuged and degassed meads (2675 centrifugal force (RCF), 6000 rpm, 10 min) were diluted in the volumetric ratio of 1:9. The analysis was carried out in accordance with the methodology presented in the studies by Kawa-Rygielska et al. [21]. The following parameters of measurements were applied: injection volume, 20 μL; elution temperature, 60 °C; flow rate, 0.6 mL/min; mobile phase, 0.005 M H_2_SO_4_; and thermostat refractometric detector at 50 °C. Concentrations of compounds were determined based on a five-point calibration curve integrated in Chromax 10.0 software by Pol-Lab (Wilkowice, Poland).

#### 3.3.3. Phenolic Compound Analysis

##### Determination of Total Polyphenols Content

The total polyphenolic content of the meads was determined using Folin–Ciocalteu’s phenol reagent (F-C) [42]. Detailed analysis is presented in the publication by Kawa-Rygielska et al. [21]. The absorbance at 765 nm was measured after 1 h, and the results are expressed as mg of gallic acid equivalents (GAE) per liter of mead. Data are expressed as the mean value for three measurements. 

##### Antioxidative Activity

The antiradical and antioxidant activity was determined using DPPH^•^ and ABTS assay [43,44,45] and also with the FRAP method based on the reduction of ferric 2,4,6-tris(2-pyridyl)-1,3,5-triazine [Fe(III)-TPTZ] to the ferrous complex at low pH, followed by spectrophotometric analysis [46]. All tests were carried out in accordance with the methodology contained in the article by Kawa-Rygielska et al. [21]. The data are expressed as Trolox equivalent (TE) of antioxidative capacity per liter of the mead (mmol TE/L). All measurements were performed in triplicate.

##### Quantification of Iridoids and Polyphenols by HPLC-PDA

The analysis was previously described by Kucharska et al. [15]. The high-performance liquid chromatography photodiode array detection method HPLC-PDA analysis was performed using a Dionex (Germering, Germany) system equipped with an Ultimate 3000 diode array detector, LPG-3400A quaternary pump, EWPS-3000SI autosampler, and TCC-3000SD thermostated column compartment, and controlled by Chromeleon v.6.8 software (Thermo Scientific Dionex, Sunnyvale, CA, USA). The analysis was based on the methodology of the publication by Kawa-Rygielska et al. [21]. Iridoids were detected at 245 nm, phenolic acid at 320 and 280 nm, flavonols at 360 nm, anthocyanins at 520 nm, and HMF at 280 nm. Loganic acid and cornuside were expressed as loganic acid, loganin and sweroside as loganin, gallic acid and gallic acid derivative as gallic acid, ellagic acid and ellagic acid derivative as ellagic acid, caffeoylquinic acids as 5-*O*-caffeoylquinic acid, *p*-coumaric acid and *p*-coumaric acid derivatives as *p*-coumaric acid, quercetin 3-*O*-glucuronide as quercetin 3-*O*-glucoside, aromadendrin 7-*O*-glucoside and kaempferol 3-*O*-galactoside as kaempferol 3-*O*-glucoside, and anthocyanins as cyanidin 3-*O*-glucoside. The results are expressed as mg per liter of the mead.

### 3.4. Statistics

Mean deviations are shown in tables. Selected data were processed using Statistica 13.5 software (StatSoft, Tulsa, OK, USA) for calculating a one-factor analysis of variance (ANOVA) with significance level α = 0.05. Differences between means were tested using the Duncan test (*p* < 0.05).

## 4. Conclusions

The study showed the possibility of using the analyzed cultivars of Cornelian cherry for the manufacture of fruit meads with health benefits. Red juice of the “Podolski” variety greatly enriched mead with compounds with strong antioxidant activity. The addition of Cornelian cherry allowed us to obtain a new alcoholic drink rich in iridoids. The content of active compounds in meads depends on the variety of Cornelian cherry fruits that is added to their production. The red fruits significantly increase the content of these compounds in the final product. Cornelian cherry mead as a fermented beverage can be a good alternative to red grape wines, if it is consumed regularly but with restraint.

## Figures and Tables

**Table 1:** Basic physicochemical characteristics and composition of meads with different types of Cornelian cherry juices at particular stages of the manufacture process.

Mead Type	Yeast	Stage of the Process	Glucose	Fructose	Ethanol	Glycerol	Acetic Acid	pH
			(g/L)	(g/L)	(g/L)	(g/L)	(g/L)	
MW ^1^		W ^2^	132.5 ± 0.48 ^d5^	193.2 ± 1.12 ^d^	nd ^4^	nd	nd	3.79 ± 0.01 ^b^
	SF ^3^	F	6.6 ± 0.79 ^o^	20.8 ± 0.98 ^s^	107.1 ± 0.01 ^c^	7.2 ± 0.42 ^i^	1.3 ± 0.15 ^a^	3.47 ± 0.01 ^c^
		A	6.0 ± 1.1 ^d^	13.5 ± 0.86 ^t^	117.8 ± 0.03 ^a^	7.8 ± 0.96 ^h^	1.3 ± 0.19 ^a^	3.38 ± 0.00 ^f^
	SM	F	8.1 ± 0.23 ^n^	46.5 ± 1.03 ^o^	85.8 ± 0.01 ^k^	7.7 ± 0.37 ^h^	1.1 ± 0.63 ^bc^	3.46 ± 0.01 ^c^
		A	3.4 ± 0.98 ^r^	29.6 ± 2.89 ^r^	104.2 ± 0.00 ^g^	8.3 ± 0.22 ^g^	1.2 ± 0.48 ^ab^	3.43 ± 0.02 ^d^
MY		W	150.8 ± 1.02 ^a^	210.5 ± 0.76 ^c^	nd	nd	nd	3.23 ± 0.01 ^i^
	SF	F	23.6 ± 0.66 ^k^	57.1 ± 3.15 ^k^	99.8 ± 0.65 ^i^	8.7 ± 0.30 ^e^	1.0 ± 0.36 ^cd^	3.31 ± 0.03 ^h^
		A	19.7 ± 0.65 ^l^	50.1 ± 1.14 ^m^	105.9 ± 0.04 ^d^	8.9 ± 0.31 ^de^	1.0 ± 0.41 ^cd^	2.84 ± 0.00 ^k^
	SM	F	35.8 ± 0.53 ^e^	97.0 ± 0.65 ^e^	73.6 ± 0.04 ^m^	8.7 ± 0.36 ^e^	nd	3.23 ± 0.02 ^i^
		A	29.0 ± 0.61 ^g^	88.3 ± 0.66 ^h^	85.8 ± 0.03 ^k^	9.2 ± 0.24 ^c^	0.8 ± 0.21 ^e^	2.89 ± 0.01 ^j^
MC		W	150.5 ± 0.12 ^b^	222.1 ± 0.54 ^a^	nd	nd	nd	3.40 ± 0.01 ^e^
	SF	F	19.5 ± 1.35 ^ł^	53.1 ± 0.24 ^l^	104.7 ± 0.02 ^f^	9.7 ± 0.04 ^b^	1.1 ± 0.19 ^bc^	3.33 ± 0.01 ^g^
		A	19.1 ± 0.02 ^m^	45.9 ± 0.00 ^p^	105.5 ± 0.01 ^e^	9.7 ± 0.25 ^b^	1.1 ± 0.14 ^bc^	3.92 ± 0.02 ^a^
	SM	F	34.7 ± 0.43 ^f^	94.7 ± 0.69 ^f^	69.0 ± 0.02 ^n^	8.5 ± 0.69 ^f^	nd	3.33 ± 0.00 ^g^
		A	26.5 ± 0.48 ^i^	87.8 ± 1.74 ^i^	88.7 ± 0.03 ^j^	9.3 ± 0.74 ^c^	0.9 ± 0.96 ^de^	3.92 ± 0.00 ^a^
MR		W	149.7 ± 0.54 ^c^	217.8 ± 0.98 ^b^	nd	nd	nd	3.34 ± 0.01 ^h^
	SF	F	24.2 ± 0.65 ^j^	52.3 ± 0.44 ^ł^	101.3 ± 0.86 ^h^	9.1 ± 0.56 ^c^	1.1 ± 0.74 ^bc^	3.31 ± 0.02 ^h^
		A	19.6 ± 0.01 ^lł^	48.8 ± 0.45 ^n^	111.8 ± 0.12 ^b^	10.0 ± 0.40 ^a^	1.1 ± 0.94 ^bc^	2.89 ± 0.01 ^j^
	SM	F	34.6 ± 0.18 ^f^	90.6 ± 0.64 ^g^	75.0 ± 0.04 ^ł^	8.9 ± 1.36 ^d^	nd	3.23 ± 0.01 ^i^
		A	28.4 ± 0.14 ^h^	83.6 ± 0.71 ^j^	83.9 ± 0.04 ^l^	9.2 ± 0.32 ^c^	nd	2.89 ± 0.00 ^j^

^1^ MW, mead without any addition; MY, mead with yellow juice; MC, mead with coral juice; MR, mead with red juice; ^2^ W, wort before fermentation; F, mead after fermentation; A, mead after aging; ^3^ SF, yeast *S. bayanus* Safspirit Fruit; SM, yeast *S. cerevisiae* Safspirit Malt; ^4^ nd, not detected; ^5^ Values are expressed as the mean (*n =* 3) ± standard deviation. Mean values with different letters (a, b, c, etc.) within the same line are statistically different (*р* < 0.05).

**Table 2:** Total polyphenols content and antioxidative activity of meads (MW, MY, MC, MR) at particular technological stages of their manufacture process: wort (W), meads after fermentation (F), meads after aging (A).

Mead Type	Yeast	Stage of the Process	Total Polyphenols (mg GAE/L)	DPPH^•^ Assay (mmol TE/L)	ABTS^⁺•^Assay (mmol TE/L)	FRAP Assay (mmol TE/L)
MW ^1^		W ^2^	18.0 ± 0.23 ^j4^	0.5 ± 0.10 ^i^	0.5 ± 0.13 ^i^	1.0 ± 0.13 ^e^
	SF ^3^	F	11.0 ± 1.01 ^j^	0.3 ± 0.09 ^i^	0.2 ± 0.21 ^i^	1.1 ± 0.01 ^e^
		A	14.0 ± 0.50 ^j^	0.2 ± 0.02 ^i^	0.3 ± 0.01 ^i^	1.0 ± 0.00 ^e^
	SM	F	5.0 ± 0.14 ^j^	0.5 ± 0.24 ^i^	0.4 ± 0.05 ^i^	1.0 ± 0.01 ^e^
		A	290.0 ± 1.25 ^j^	0.1 ± 0.04 ^i^	0.2 ± 0.12 ^i^	1.0 ± 0.24 ^e^
MY		W	617.6 ± 1.01 ^f^	5.3 ± 0.14 ^ef^	5.5 ± 0.12 ^ef^	5.4 ± 0.23 ^d^
	SF	F	484.9 ± 1.08 ^g^	4.8 ± 0.01 ^fg^	5.1 ± 0.43 ^fg^	6.0 ± 0.05 ^cd^
		A	378.8 ± 0.07 ^i^	4.2 ± 0.06 ^h^	4.1 ± 0.23 ^h^	5.6 ± 0.10 ^h^
	SM	F	596.8 ± 1.19 ^f^	4.6 ± 0.44 ^gh^	4.0 ± 0.24 ^gh^	5.6 ± 0.04 ^cd^
		A	399.7 ± 1.00 ^hi^	4.4 ± 0.12 ^gh^	4.0 ± 0.03 ^h^	5.2 ± 0.07 ^h^
MC		W	1006.0 ± 7.94 ^b^	6.5 ± 0.16 ^g^	8.7 ± 0.94 ^a^	10.0 ± 3.12 ^a^
	SF	F	518.2 ± 2.04 ^g^	7.2 ± 1.23 ^a^	6.1 ± 0.19 ^cde^	5.0 ± 0.69 ^d^
		A	613.6 ± 0.15 ^f^	5.4 ± 0.04 ^de^	6.0 ± 0.15 ^cde^	5.3 ± 0.37 ^d^
	SM	F	596.8 ± 1.19 ^f^	5.5 ± 0.33 ^de^	6.6 ± 0.76 ^bcd^	8.0 ± 0.36 ^b^
		A	423.1 ± 4.74 ^h^	5.4 ± 0.14 ^de^	5.8 ± 0.30 ^def^	6.2 ± 0.14 ^cd^
MR		W	1219.8 ± 2.36 ^a^	7.3 ± 0.11 ^a^	9.4 ± 0.69 ^a^	10.4 ± 0.01 ^a^
	SF	F	808.4 ± 2.18 ^e^	6.6 ± 0.17 ^b^	6.9 ± 1.47 ^bc^	8.1 ± 0.15 ^bc^
		A	774.3 ± 1.09 ^e^	5.9 ± 0.06 ^cd^	6.4 ± 0.21 ^bcd^	8.1 ± 0.87 ^b^
	SM	F	946.1 ± 2.32 ^c^	6.3 ± 0.08 ^bc^	8.6 ± 0.15 ^a^	6.9 ± 0.21 ^bc^
		A	898.7 ± 1.05 ^d^	6.2 ± 0.08 ^bc^	7.1 ± 0.16 ^b^	8.2 ± 0.11 ^b^

^1^ MW, mead without any addition; MY, mead with yellow juice; MC, mead with coral juice; MR, mead with red juice; ^2^ W, wort before fermentation; F, mead after fermentation; A, mead after aging; ^3^ SF, yeast *S. bayanus* Safspirit Fruit; SM, yeast *S. cerevisiae* Safspirit Malt; ^4^ Values are expressed as the mean (*n =* 3) ± standard deviation. Mean values with different letters (a, b, c, etc.) within the same line are statistically different (*р* < 0.05).

**Table 3:** Iridoids and phenolic acids content (mg/L) in mead samples (MW, MY, MC, MR) at different stages of their production: worts (W), samples after alcoholic fermentation (F), and meads after aging (A), fermented with different yeast: *S. bayanus* Safspirit Fruit (SF), *S. cerevisiae* Safspirit Malt (SM).

Mead Type	Yeast	Stage of the Process	LA ^4^	S + Lo	Co	Total Iridoids	GA	GAd	Total GA	EA	EAd	Total EA	Total HBA	*p*-CuAd 4.0 min	*p*-CuAd 4.3 min	CQA	*p*-CuA 9.5 min	Total HCA
MW ^1^	W ^2^		nd ^5^	nd	nd	nd	nd	nd	nd	nd	nd	nd	nd	nd	nd	0.3	0.1	0.5
	SF ^3^	F	nd	nd	nd	nd	nd	nd	nd	nd	nd	nd	nd	nd	nd	nd	0.1	0.1
		A	nd	nd	nd	nd	nd	nd	nd	nd	nd	nd	nd	nd	nd	nd	0.1	0.1
	SM	F	nd	nd	nd	nd	nd	nd	nd	nd	nd	nd	nd	nd	nd	nd	0.1	0.1
		A	nd	nd	nd	nd	nd	nd	nd	nd	nd	nd	nd	nd	nd	nd	nd	nd
Y			100.6	15.1	11.9	127.6	3.3	nd	3.3	0.2	nd	0.3	3.6	1.0	0.3	5.8	0.2	7.4
MY	W		64.4	2.6	4.2	71.2	0.8	nd	0.8	0.1	nd	0.1	0.9	0.3	0.1	2.4	0.1	2.9
	SF	F	65.1	2.5	2.6	70.1	1.3	1.5	2.8	0.3	0.2	0.3	3.1	0.2	0.1	1.6	0.1	2.1
		A	48.8	2.1	3.1	54.0	1.5	1.2	2.8	0.2	0.1	0.2	2.9	0.1	0.1	1.0	0.1	1.4
	SM	F	65.3	2.5	2.7	70.5	1.5	1.6	3.1	0.3	0.2	0.3	3.3	0.3	0.1	1.6	0.1	2.0
		A	54.8	2.4	2.7	60.0	2.3	1.5	3.8	0.2	0.1	0.2	4.1	0.2	0.2	1.2	0.1	1.6
C			171.5	24.3	16.6	212.4	4.5	nd	4.5	0.5	nd	0.5	5.1	1.1	0.4	8.1	0.3	9.9
MC	W		88.3	3.6	4.8	96.8	1.1	nd	1.1	0.1	nd	0.1	1.2	0.3	0.1	3.0	0.2	3.5
	SF	F	89.0	3.0	3.1	95.0	2.5	1.2	3.7	0.2	nd	0.2	3.8	0.2	0.1	2.4	0.1	2.9
		A	77.8	3.2	2.8	83.8	1.5	1.6	3.1	0.2	0.2	0.4	3.5	0.2	0.2	2.0	0.2	2.9
	SM	F	86.7	2.9	3.3	92.9	2.5	1.3	3.8	0.2	nd	0.2	4.0	0.2	0.1	2.4	0.2	2.5
		A	76.4	3.0	2.6	82.0	1.2	1.9	3.1	0.2	0.2	0.3	3.4	0.2	0.2	1.8	0.1	2.3
R			128.7	34.5	14.5	177.7	5.1	nd	5.1	0.6	nd	0.6	5.7	0.5	0.2	7.7	0.3	8.8
MR	W		51.7	8.8	4.1	64.6	1.2	nd	1.2	0.1	nd	0.1	1.4	0.2	0.1	2.7	0.2	3.1
	SF	F	54.0	3.9	2.6	60.6	1.8	1.6	3.4	0.2	nd	0.2	3.6	0.1	nd	1.8	0.1	2.1
		A	48.8	3.9	2.8	51.4	1.2	1.3	2.9	0.2	0.2	0.4	3.3	0.1	0.1	1.4	0.1	1.7
	SM	F	52.5	2.6	nd	59.3	1.9	1.8	3.2	0.2	nd	0.2	3.4	0.1	nd	1.8	0.2	2.1
		A	48.8	2.9	nd	51.7	1.0	2.0	3.0	0.2	0.1	0.4	3.4	0.1	0.1	1.4	0.1	1.8

^1^ MW, mead without any addition; MY, mead with yellow juice; MC, mead with coral juice; MR, mead with red juice; ^2^ W, wort before fermentation; F, mead after fermentation; A, mead after aging; Y, yellow juice; C, coral juice; R, red juice; ^3^ SF, yeast *S. bayanus* Safspirit Fruit; SM, yeast *S. cerevisiae* Safspirit Malt; ^4^ LA, loganic acid; S, Sweroside; Lo, Loganin; Co, cornuside; GA, gallic acid; GAd, gallic acid derivative; EA, ellagic acid, EAd, ellagic acid derivative; *p*-CuAd, *p*-coumaric acid derivative; CQA, 5-*O*-caffeoylquinic acid; *p*-CuA, *p*-coumaric acid; HBA, hydroxybenzoic acids; HCA, hydroxycinnamic acids; ^5^ nd, not detected.

**Table 4:** Flavonols, anthocyanins, and hydroxymethylfurfural content (mg/L) in mead samples (MW, MY, MC, MR) at different stages of their production: worts (W), samples after alcoholic fermentation (F), and meads after aging (A), fermented with different yeast: *S. bayanus* Safspirit Fruit (SF), *S. cerevisiae* Safspirit Malt (SM).

Mead Type	Yeast	Stage of the Process	Q-3-glcr ^4^	Kf-3-gal	A-7-glc	Total Flavonols	Df-3-gal	Cy-3-gal	Cy-3-rob	Pg-3-gal	Pg-3-rob	Total Antocyjanins	HMF
MW ^1^	W ^2^		nd ^5^	nd	nd	nd	nd	nd	nd	nd	nd	nd	0.45
	SF ^3^	F	nd	nd	nd	nd	nd	nd	nd	nd	nd	nd	0.06
		A	nd	nd	nd	nd	nd	nd	nd	nd	nd	nd	0.05
	SM	F	nd	nd	nd	nd	nd	nd	nd	nd	nd	nd	0.07
		A	nd	nd	nd	nd	nd	nd	nd	nd	nd	nd	0.04
Y			2.42	nd	nd	2.42	nd	nd	nd	nd	nd	nd	7.37
MY	W		0.55	nd	nd	0.55	nd	nd	nd	nd	nd	nd	2.86
	SF	F	0.59	nd	nd	0.59	nd	nd	nd	nd	nd	nd	2.08
		A	0.40	nd	nd	0.40	nd	nd	nd	nd	nd	nd	1.41
	SM	F	0.61	nd	nd	0.61	nd	nd	nd	nd	nd	nd	2.04
		A	0.50	nd	nd	0.50	nd	nd	nd	nd	nd	nd	1.59
C			3.06	nd	nd	3.06	nd	0.27	nd	1.79	nd	2.06	9.85
MC	W		0.81	nd	nd	0.81	nd	0.06	nd	0.47	nd	0.53	3.51
	SF	F	0.79	nd	nd	0.79	nd	0.02	nd	0.17	nd	0.19	2.88
		A	0.70	nd	nd	0.70	nd	nd	nd	nd	nd	nd	2.54
	SM	F	0.80	nd	nd	0.80	nd	0.02	nd	0.15	nd	0.17	2.91
		A	0.69	nd	nd	0.69	nd	nd	nd	0.02	nd	0.02	2.25
R			2.78	1.63	1.45	5.86	0.59	8.15	3.30	15.69	2.87	30.61	8.84
MR	W		0.71	0.46	0.39	1.56	0.14	2.09	0.91	4.09	0.72	7.94	3.08
	SF	F	0.69	0.47	0.42	1.58	0.05	0.72	0.39	1.43	0.34	2.93	2.10
		A	0.55	0.33	0.47	1.35	nd	0.03	0.02	1.51	0.02	0.13	1.74
	SM	F	0.68	0.44	0.41	1.53	0.05	0.76	0.40	0.06	0.34	3.05	2.12
		A	0.59	0.34	0.48	1.41	nd	0.03	0.02	0.07	0.02	0.15	1.76

^1^ MW, mead without any addition; MY, mead with yellow juice; MC, mead with coral juice; MR, mead with red juice; ^2^ W, wort before fermentation; F, mead after fermentation; A, mead after aging; Y, yellow juice; C, coral juice; R, red juice; ^3^ SF, yeast *S. bayanus* Safspirit Fruit; SM, yeast *S. cerevisiae* Safspirit Malt; ^4^ Q-3-glcr, quercetin 3-*O*-glucuronide; Kf-3-gal, kaempferol 3-*O*-galactoside; A-7-glc, aromadendrin 7-glucoside; Df-3-gal, delphinidin 3-*O*-galactoside; Cy-3-gal, cyjanidin 3-*O*-galactoside; Cy-3-rob, cyanidin 3-*O*-robinobioside; Pg-3-gal, pelargonidin 3-*O*-galactoside; Pg-3-rob, pelargonidin 3-*O*-robinobioside; HMF, hydroxymethylfurfural; ^5^ nd, not detected.

**Table 5:** Description and symbols of cherry fruit juices used in the experiment, worts, and meads obtained in the study.

Symbol	Description	
Y	cherry fruit juices	juice from yellow fruit of Cornelian cherry
C		juice from coral fruit of Cornelian cherry
R		juice from red fruit of Cornelian cherry
F	mead production steps	fermentation
A		aging
W	wort	control wort
W-Y		wort with the addition of yellow Cornelian cherry juice
W-C		wort with the addition of coral Cornelian cherry juice
W-R		wort with the addition of red Cornelian cherry juice
MW	mead	control mead
MY		mead with the addition of yellow Cornelian cherry juice
MC		mead with the addition of coral Cornelian cherry juice
MR		mead with the addition of red Cornelian cherry juice

## Supplementary Materials

The following are available online, Figure S1: HPLC-DAD chromatograms (245 nm, 360 nm, 520 nm) of compounds of red cornelian cherry juice (GA, gallic acid; LA, loganic acid; S, Sweroside; Lo, Loganin; Co, cornuside; EA, ellagic acid; Df-3-gal, delphinidin 3-*O*-galactoside; Cy-3-gal, cyjanidin 3-*O*-galactoside; Cy-3-rob, cyjanidin 3-*O*-robinobioside; Pg-3-gal, pelargonidin 3-*O*-galactoside; Pg-3-rob, pelargonidin 3-*O*-robinobioside; Cy, cyjanidin; Pg, pelargonidin; A-7-glc, aromadendrin 7-glucoside; Q-3-glcr, quercetin 3-*O*-glucuronide; Kf-3-gal, kaempferol 3-*O*-galactoside).
