# Supplementary material for: Characteristics of Biologically Active Compounds in Cornelian Cherry Meads
Source: Molecules. 2018 Aug 14;23(8):2024. doi: 10.3390/molecules23082024 (PMC6222799; doi:10.3390/molecules23082024)
Supplement: Supplementary file 1 [file molecules-23-02024-s001.pdf]

molecules

Characteristics of biologically active compounds in Cornelian cherry meads

Kinga Adamenko[1,*], Joanna Kawa-Rygielska[1], Alicja Z. Kucharska[2], Narcyz Piórecki[3,4]

[1]Department of Fermentation and Cereals Technology, Faculty of Food Science, Wroclaw University of Environmental and Life Sciences, Chełmońskiego 37, 51-630 Wrocław, Poland; joanna.kawa-rygielska@upwr.edu.pl
[2]Department of Fruit, Vegetable and Plant Nutraceutical Technology, Faculty of Food Science, Wroclaw University of Environmental and Life Sciences, Chełmońskiego 37, 51-630 Wrocław, Poland; alicja.kucharska@upwr.edu.pl
[3]Arboretum and Institute of Physiography in Bolestraszyce, 37-700 Przemyśl, Poland; narcyz360@gmail.com
[4]Faculty of Physical Education, University of Rzeszów, Towarnickiego 3, 35-959 Rzeszów, Poland
[*]Correspondence: kinga.adamenko@upwr.edu.pl; Tel.: +48-71-323-9417

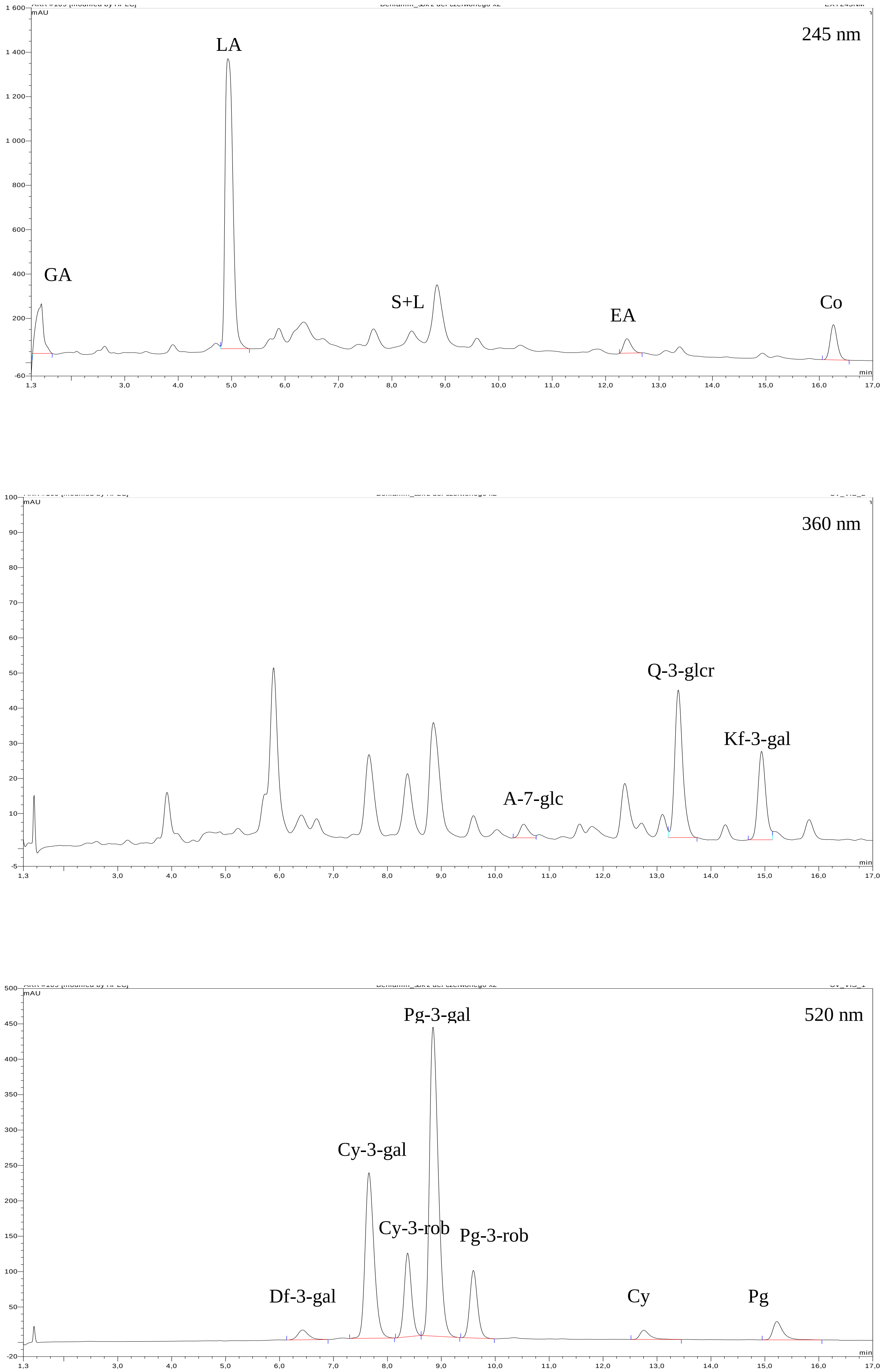
245 nm
LA
GA
S+L
EA
Co
360 nm
Q-3-glcr
Kf-3-gal
A-7-glc
520 nm
Pg-3-gal
Cy-3-gal
Cy-3-rob
Pg-3-rob
Df-3-gal
Cy
Pg
mAU
min

Fig. S1. HPLC-DAD chromatograms (245 nm, 360 nm, 520 nm) of compounds of red cornelian cherry juice (GA, gallic acid; LA, loganic acid; S, Sweroside; Lo, Loganin; Co, cornuside; EA, ellagic acid; Df-3-gal, delphinidin 3-O-galactoside; Cy-3-gal, cyjanidin 3-O-galactoside; Cy-3-rob, cyjanidin 3-*O*-robinobioside; Pg-3-gal, pelargonidin 3-*O*-galactoside; Pg-3-rob, pelargonidin 3-*O*-robinobioside; Cy, cyjanidin; Pg, pelargonidin; A-7-glc, aromadendrin 7-glucoside; Q-3-glcr, quercetin 3-O-glucuronide; Kf-3-gal, kaempferol 3-O-galactoside).
